# *Lactobacillus reuteri* Alleviates Gastrointestinal Toxicity of Rituximab by Regulating the Proinflammatory T Cells *in vivo*

**DOI:** 10.3389/fmicb.2021.645500

**Published:** 2021-10-12

**Authors:** Binyan Zhao, Bailing Zhou, Chunyan Dong, Rui Zhang, Daoyuan Xie, Yaomei Tian, Li Yang

**Affiliations:** State Key Laboratory of Biotherapy and Cancer Center, West China Hospital, Collaborative Innovation Center of Biotherapy, Sichuan University, Chengdu, China

**Keywords:** rituximab, intestinal microbiota, *Lactobacillus reuteri*, gavage methods, gastrointestinal toxicity

## Abstract

Rituximab (RTX) is a widely used anticancer drug with gastrointestinal side effects, such as nausea, vomiting, and diarrhea. The reason for these side effects is still poorly understood. Previous studies have reported that the intestinal microbiota is associated with the occurrence of disease and the therapeutic effect of drugs. In this study, we observed mucosal damage, inflammatory cell infiltration and increased intestinal inflammatory factor expression in RTX-treated mice. RTX also changed the diversity of the intestinal microbiota in mice, and decreased abundance of *Lactobacillus reuteri* was observed in RTX-treated mice. Further experiments revealed that intragastric administration of *L. reuteri* in RTX-treated mice attenuated the intestinal inflammatory response induced by RTX and regulated the proportion of helper T (Th) cells. In conclusion, our data characterize the effect of the intestinal microbiota on RTX-induced intestinal inflammation, suggesting that modifying the gut microbiota may represent a positive strategy for managing adverse reactions.

## Introduction

Although there are many new methods for cancer treatment, drug therapy for cancer is still an important and indispensable treatment modality that is mainly based on the ability of drugs to clear cancer cells, reduce tumor growth and relieve pain. Anticancer drugs kill tumor cells by specifically recognizing surface markers on tumor cells and stimulating the immune response. For example, rituximab binds to the CD20 antigen on B-cell-derived tumor cells to initiate an immune response that mediates B-cell lysis. Possible mechanisms of B-cell lysis include complement-dependent cytotoxicity (CDC) and antibody-dependent cytotoxic (ADCC) effects. These killing effects are not specific to tumor cells and also affect B cells, T cells, and regulatory T cells. Infection and gastrointestinal toxicity often occur during the course of treatment (El Fassi et al., 2008; Agathocleous et al., 2010; Ardelean et al., 2010; Blombery et al., 2011; Sekkach et al., 2011; Lipka et al., 2016; Shahmohammadi et al., 2018; Kaegi et al., 2019), and they can treatment efficacy and patient compliance to a certain extent. However, there is currently no effective treatment to reduce gastrointestinal mucosal lesions. After proliferation and differentiation, crypt cells migrate to the villi of the small intestine to replace the exfoliated mature epithelial cells and become intestinal mucosal epithelial cells (Farrell et al., 1998; Bau et al., 2016). Some drugs kill crypt stem cells of the small intestine, damaging the absorption and barrier function of the intestinal mucosa and resulting in gastrointestinal tract toxicity and side effects. Some studies have shown that in cancer chemotherapy, the incidence of intestinal mucositis is as high as 40% (Boukhettala et al., 2009; Beutheu et al., 2014). An increasing number of studies have shown that the intestinal microbiota is involved in regulating the efficacy and toxicity of chemotherapy drugs (Li et al., 2016, 2017; Wardill and Tissing, 2017; Wilson and Nicholson, 2017; Zimmermann et al., 2019).

There are a large number of microbial communities in the intestine, including approximately 3 × 10^13^ bacteria (Sender et al., 2016). Most of them coexist with the host in a dynamic balance. However, when the health status of the host is affected, the composition of intestinal flora and the abundance of certain bacteria change. Expansion of some symbiotic bacteria may lead to more serious effects, while the reduction of some symbiotic bacteria will cause the opposite effects; for example, Routy et al. (2018) found that patients with high *Akkermansia muciniphila* abundance tend to experience improved efficacy after receiving PD-1 therapy (Zhou et al., 2018). Intestinal microbes even play an important role in the health of the host by regulating the immune response or the well-known “liver–brain–gut” axis circulation. When the tissue is damaged after infection or injury, inflammatory cells enter the intestine from the systemic circulation or intestinal cistern to restore the body’s balance (Zhou et al., 2018). Therefore, intestinal injury caused by RTX may cause an imbalance in enteric cells, which may lead to malignant inflammatory circulation.

It should be noted that a large number of inflammatory cells in the intestine can also affect the intestinal microbiota. In addition, some drugs can affect the intestinal microbiota. For example, Viaud et al. found that after 7 days of cyclophosphamide treatment, the total number of bacteria in the small intestine of mice did not decrease, but the abundance of *Lactobacillus* and *enterococci* decreased. Moreover, after drug treatment, the villi of the small intestine in mice were shortened, the intestinal barrier was damaged, and inflammatory cells had aggregated (Viaud et al., 2013). Moreover, specific gram-positive bacteria (*Lactobacillus johnsonii* and *Enterococcus hirae*) regulate the accumulation of cyclophosphamide-driven Th1 and Th17 cell responses (Viaud et al., 2013). It has been reported that CTLA4 antagonists induce T cell-mediated intestinal mucositis, which is related to changes in the intestinal flora (Viaud et al., 2013; Authors et al., 2015; Sivan et al., 2015; Vétizou et al., 2015). These studies demonstrate that homeostasis of the intestinal flora is important for the host immune system and intestinal epithelium. In our study, we identified the effect of rituximab on intestinal mucosal injury, the changes in different immune cells in mesenteric lymph nodes, and the resulting changes in intestinal microbiota, which are associated with the development of intestinal mucositis. We also found that this toxicity is attenuated by intragastric administration of specific Lactobacillus species. Our aim was to characterize these changes and examine the effects of altering the intestinal microbiota on rituximab-induced mucositis. These findings may contribute to subsequent studies on methods to attenuate the gastrointestinal toxicity of certain drugs.

## Materials and Methods

### Animal Experiments

Female BALB/c mice (6–8 weeks old) were purchased from Beijing Vital River Laboratory Animal Co., Ltd. (Beijing, China). All animals were housed in a pathogen-free environment and maintained at 22 ± 2°C under 12-h light/dark cycle conditions. All animal experiments were performed according to the guidelines approved by the Animal Protection Committee of Sichuan University (Sichuan, China, ID: 2018091815).

Two experiments were performed to investigate the relationship between rituximab toxicity and the intestinal microbiota. In the first experiment, female BALB/c mice were divided into two groups. Mice in the experimental group were intraperitoneally injected with 4 mg rituximab (Roche, 100 mg/10 ml), while mice in the control group received sterile phosphate balanced solution (PBS). In the second experiment, female BALB/c mice were randomly divided into six groups with six mice per group. Mice in the antibiotic (ATB) group, ATB + RTX group and ATB + RTX + *Lactobacillus reuteri* group were treated with metronidazole (1 g/L, Solarbio 443-48-41) supplied in the drinking water on day −7 for 5 days that was then stopped for 2 days. On day 0, mice in the RTX and ATB + RTX groups were intraperitoneally injected with rituximab at 4 mg/mouse. Mice in the RTX + *L. reuteri* and ATB + RTX + *L. reuteri* groups were simultaneously gavaged with 1 × 10^8^ CFU of *L. reuteri* in 200 μl PBS for 7 days. The other two groups (ATB and Con) were gavaged with sterile PBS. All mice were sacrificed on day 7.

### Bacterial Culture

*Lactobacillus reuteri* (ATCC 53608) was purchased from American Type Culture Collection (ATCC) and were cultured in De Man, Rogosa and Sharpe (MRS) broth medium (M264-02) at 37°C and under anaerobic conditions. The bacterial suspension was washed with sterile PBS, centrifuged (3,200*g*, 5 min) and resuspended in PBS until OD_600_ = 1, which was approximately 1 × 10^8^ colony forming units (CFUs)/ml (Jiménez-Flores et al., 2010).

### Stool Sample Collection and DNA Extraction

According to different animal experimental schemes, stool samples were collected from mice on days −7, 0, and 7 during the first experiment and immediately stored at −80°C. In the second animal experiment, fecal samples were collected from mice on days −7, −5, 0, and 7 and were immediately stored at −80°C until processing with a fecal DNA isolation kit (Foregene Co., Ltd., Sichuan, China). Samples were stored at −80°C according to the fecal DNA isolation kit manufacturer’s instructions.

### 16S Sequencing and Bioinformatics Analysis

The V4 region of 16S rDNA was amplified by Beijing Novogene Technology Co., Ltd., and sequenced on the NovaSeq6000 platform. Paired-end reads were merged using FLASH (V1.2.7)^1^ (Magoč and Salzberg, 2011), a fast and accurate analysis tool that was designed to merge paired-end reads when at least some of the reads overlapped reads generated from the opposite end of the same DNA fragment, and the splicing sequences were represented raw tags. Quality filtering of the raw tags was performed under specific filtering conditions to obtain high-quality clean tags (Bokulich et al., 2013) according to QIIME (V1.9.1).^2^ Tags were subsequently compared with the reference database (Silva database)^3^ using the UCHIME algorithm (UCHIME algorithm)^4^ (Edgar et al., 2011) to detect chimeric sequences, which were then removed (Haas et al., 2011), yielding the effective tags.

Sequence analysis was performed using Uparse software (Uparse v7.0.1001)^5^ (Edgar, 2013). Sequences with ≥97% similarity were assigned to the same OTUs.

Representative sequences for each OTU were screened for further annotation. For each representative sequence, the Silva Database (see text footnote 3) (Quast et al., 2013) was used based on the Mothur algorithm to annotate taxonomic information. To study the phylogenetic relationship of different OTUs and the difference in the dominant species in different samples (groups), multiple sequence alignment was conducted using MUSCLE software (Version 3.8.31)^6^ (Edgar, 2004).

Linear discriminant analysis (LDA) and effect size (LEfSe) analysis methods (Segata et al., 2011) used the Kruskal-Wallis test to compare the abundance of all bacterial clades between day 0 and day 7 at a predefined α of 0.05. Significantly different vectors obtained by comparing abundance among the groups were used as inputs for LDA, resulting in an effect size. Compared with traditional statistical tests, the main advantage of LEfSe is that in addition to the *p*-value, LEfSe also produces an effect size. This feature allows us to sort the results of multiple tests based on the size of the difference between groups.

### Quantitative Real-Time PCR

Quantitative real-time PCR (qPCR) was used to quantify bacterial 16S rDNA gene abundance in feces samples. Primers targeting the rDNA gene of *L. reuteri* (F: ACCGAGAACACCGCGTTATTT, R: CATAACTTAACCTAAACAATCAAAGATTGTCT) (Rothhammer et al., 2016) and primers targeting β-actin (F: CCCAGGCATTGCTGACAGG, R: TGGAAGGTGGACAGTGA GGC) were used.

Total RNA was extracted from cells or small intestine segments of mice using a kit (Foregene, China) and was quantified using a Nanodrop ND-1000 spectrophotometer (Thermo Scientific). According to the manufacturer’s instructions, cDNA was synthesized using a PrimeScript RT Kit with gDNA Eraser (Vazyme, China). Transcription levels of TNF-α, IL-1β, IL-6, IL-10, claudin-1, and β-actin were analysed by SYBR Green PCR Master Mix (Vazyme, China) on a CFX96 real-time system (Bio-Rad). The relative expression was calculated by ΔCT method. The sequences of the gene-specific primers were as follows: TNF-α (F: CCCAGGGACCTCTCTCTAATC, R: ATGGGCTACAGGCTTGTCACT); IL-1β (F: ATCTCGCAGCA GCACATCAA, R: ACGGGAAAGACACAGGTAGC); IL-6 (F: CCAGTTGCCTTCTTGGGACT, R: GTCTCCTCTCCGGACTT GTG); IL-10 (F: CATCGATTTCTTCCCTGTGAA, R: TCTTGGAGCTTATTAAAGGCATTC); and claudin-1 (F: TGCCCCAGTGGAAGATTTACT, R: CTTTGCGAAACG CAGGACAT).

Quantitative real-time PCR was performed on a CFX96 Real-time system (Bio-Rad) under the following cycling conditions: 95°C for 30 s, 35 cycles at 95°C for 10 s, and 60°C for 30 s. Then, the default melting curve acquisition program was used.

### Hematoxylin and Eosin Staining for Histological Analyses

The mice were sacrificed 1 week after RTX treatment. Ileum and colon samples were fixed in 4% PFA for at least 48 h. Subsequently, the tissues were cut into small pieces, embedded in paraffin and cut into 4-μm sections that were stained with hematoxylin and eosin (H&E) and scored by pathologists. In the semi-quantitative analysis of histology, villus atrophy, necrosis, and inflammatory cell infiltration scores were as follows: 0 (no injury), 1 (mild injury), 2 (moderate injury), and 3 (severe injury). The lengths of 10 intact and well-oriented villi were measured using ImageJ software (Yu et al., 2019).

### Flow Cytometry Experiments

Monoclonal antibodies against mouse CD3E, CD4, Foxp3, IL-4, CD19, IL-10, IFN-γ, IL-17A, CD11b, CD11c, F4/80, CD206, and CD16/32 were purchased from BD Biosciences. All mice were sacrificed at the end of the experiment. Mesenteric lymph nodes (MLNs) and spleens were crushed in a cell filter (75 μm nylon; BD Falcon), and the cell suspension was treated with erythrolysis buffer (Beyotime Biotechnology) at room temperature for 5 min. Then, the cells were washed three times and suspended in PBS. For surface antibody staining, the cell suspension was combined with various antibodies and incubated in the dark at 4°C for 30 min. To stain for Foxp3, IL-4, IL-10, IL-17A, and IFN-γ, the cells were fixed in 4% neutral paraformaldehyde after surface marker staining, treated with 0.5% Triton-100 (PBS dilution) at 4°C for 20 min, washed again, and then incubated with corresponding antibodies. Flow cytometry (FACSCalibur; BD Biosciences) was used to obtain the cell events, and the data were analysed with NovoExpress software.

### Cell Culture and Stimulation With *Lactobacillus reuteri in vitro*

The mesenteric lymph node (MLN) cells of mice were cultured at 37°C and 5% CO_2_. Ten percent fetal bovine serum (Gibco, United States) was added to RPMI 1640 cell culture medium. For *L. reuteri*’s anti-inflammatory test, MLN cells were cultured in a 6-well plate at a density of 2 × 10^6^ cells per well and then co-incubated with *L. reuteri* (infection multiple = 10, 2 × 10^6^ cells and 2 × 10^7^ CFU) in the presence or absence of LPS (10 ng/mL, PrimeGene) (Chae, 2018) for 4 h. After incubation, cells were collected for gene expression analysis.

### Statistical Analysis

All data were statistically analysed using GraphPad Prism 8 and are shown as the mean value ± SEM of the mean. Statistical significance was evaluated at a level of *P* < 0.05 using unpaired two-way analysis of variance (ANOVA) or *t*-test analysis, ^∗^ indicated significance at *p* < 0.05, ^∗∗^*p* < 0.01, ^∗∗∗^*p* < 0.001, ^****^P < 0.0001, respectively.

## Results

### Rituximab Induces Intestinal Mucositis in Mice

**FIGURE 1:**
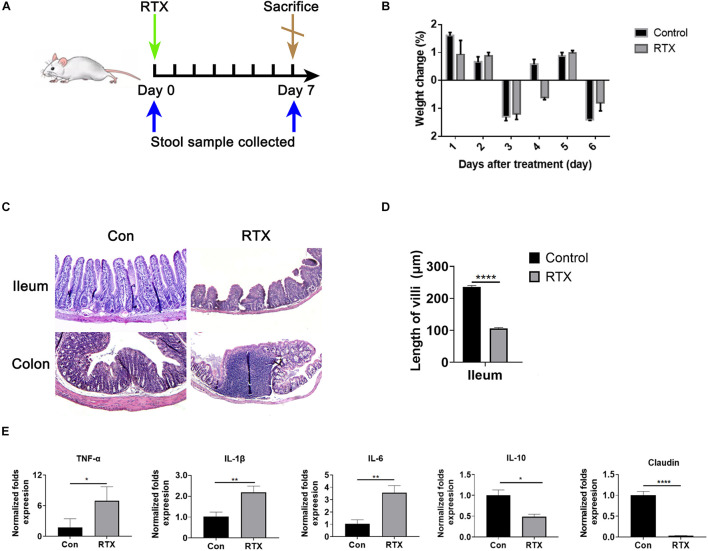
Rituximab (RTX) causes marked intestinal mucositis. **(A)** Schematic diagram of RTX administration and stool sample collection. BALB/c mice were intraperitoneally injected with RTX or normal saline on day 0 and then sacrificed on day 7. Feces were collected before and after treatment. **(B)** From day 0 to day 7, the body weights of mice in the control and RTX treatment groups were changed. **(C)** H&E staining of the ileum and colon (magnification, 40×). **(D)** Villus length of ileum and colon. **(E)** Expression levels of TNF-α, IL-1β, IL-6, IL-10, and claudin in the ileum of mice treated with RTX or normal saline. Data are expressed as the mean ± SEM. *p < 0.05, **p < 0.01, ****P < 0.0001.

To investigate the intestinal toxicity of RTX, mice were treated with a single dose of RTX (4 mg/mouse, i.p.) for 7 days (Figure 1A). Compared with the control group, RTX caused significant weight loss in mice (Figure 1B). In contrast to the control group, villous atrophy and intestinal epithelial injury of mice in the RTX treatment group were obvious, as shown in Figure 1C. Moreover, obvious inflammatory cell infiltration was apparent in the colon tissue of RTX-treated mice (Figure 1C). Compared with the control group, the intestinal mucositis score (Supplementary Figure 2) in the RTX group was significantly increased (*P* < 0.001; Figure 1D). These results suggest that RTX treatment causes intestinal damage and inflammatory cell infiltration. To further verify the intestinal inflammatory response induced by RTX, qPCR was used to assess the expression of inflammatory markers in the ileum. We observed that expression levels of the proinflammatory cytokines TNF-α, IL-1β, and IL-6 in the ileum of RTX-treated mice were significantly increased, while expression of IL-10 was significantly decreased (Figure 1E), the colon inflammatory factor data in the Supplementary Figure 3. Claudin-1 is a tight junction transmembrane protein that constitutes tight junctions between cells. It functions mainly to regulate the permeability of the barrier structure. The stability of tight junctions is related to the complex interactions between claudin proteins and other tight junction proteins. Our results showed that compared with the control group, the expression of claudin in the intestinal tissue of RTX-treated mice was significantly decreased (*P* < 0.0001; Figure 1E), which may increase intestinal permeability, cause bacterial migration to the site of intestinal injury, and further promote the local inflammatory response.

### Rituximab Induces Alterations in T Cell Subsets

**FIGURE 2:**
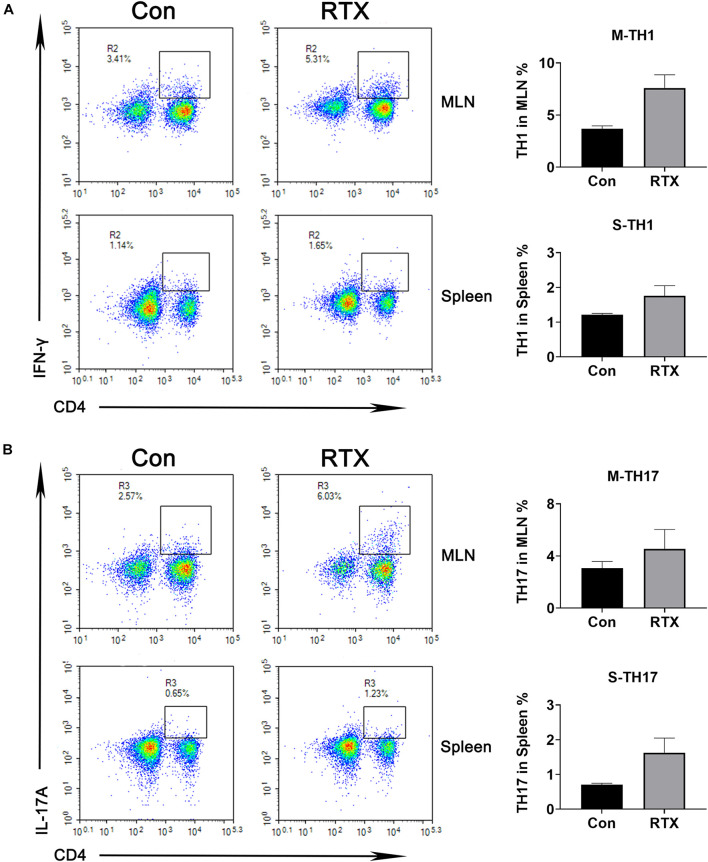
Changes in helper T (Th) cell subsets are induced by RTX. The percentages of Th1 and Th17 cells in the spleen and MLN were detected by flow cytometry. Data are expressed as the mean ± SEM. *p* < 0.05.

We observed that compared with the control group, the infiltration of CD4^+^ dendritic cells in intestinal tissues were significantly increased after RTX treatment (Supplementary Figure 1), and CD4^+^ cells were subdivided into subsets of T helper (Th), such as Th1, Th2, Th9, Th17, regulatory T (Treg) cell, and follicular helper T cell subtypes. Our results indicated that compared with the control group, levels of Th1 (CD4^+^/IFN-γ^+^) and Th17 cells (CD4^+^/IL-17A^+^) in the MLN of RTX-treated mice were significantly increased (Figure 2). Th1 cells promote the cell-mediated inflammatory response by inducing the activation of macrophages, NK cells, and B cells (Carrasco and Fernández-Bañares, 2017). Most of the effector capacity of Th17 cells comes from the fact that IL-17, together with TNF-α, strongly promote inflammation by inducing the expression of adhesion molecules, proinflammatory cytokines (e.g., IL-6, GM-CSF, and G-CSF), chemokines, prostaglandin E2 and matrix metalloproteinases (Kumawat et al., 2013; Rauber et al., 2017). Th17 cells respond to infection by extracellular bacteria and fungi. Elevated Th17 cells have been found in patients with atopic dermatitis, Crohn’s disease, psoriasis, and multiple sclerosis.

In addition, macrophages can adopt specialized functional phenotypes in response to various signals, known as classic activated macrophages (CD11b^+^F4/80^+^) and selectively activated macrophages (CD11b^+^CD206^+^) (Mantovani et al., 2002; Sica and Mantovani, 2012). CD11b^+^F4/80^+^ macrophages secrete proinflammatory cytokines, present antigens, promote the Th1 response, and mainly participate in the positive immune response. In contrast, CD11b^+^cd206^+^ macrophages play an immunomodulatory role by producing inhibitory cytokines, such as IL-10 or TGF-β, and downregulating the immune response (Sica and Bronte, 2007; Martinez and Gordon, 2014; Acharya et al., 2020). Compared with the control group, the content of CD11b^+^F4/80^+^ macrophages in the spleen and MLN were increased after RTX treatment, while CD11b^+^CD206^+^ macrophages were slightly decreased (Supplementary Figure 1). In addition, the ratio of CD11b^+^F4/80^+^/CD11b^+^CD206^+^ cells in the RTX treatment group was significantly increased, suggesting that RTX promotes the polarization of inflammatory macrophages induced by CD11b^+^F4/80^+^ cells in mice, inducing intestinal inflammation (Supplementary Figure 1).

### Rituximab Induces Changes in Intestinal Microbial Components

**FIGURE 3:**
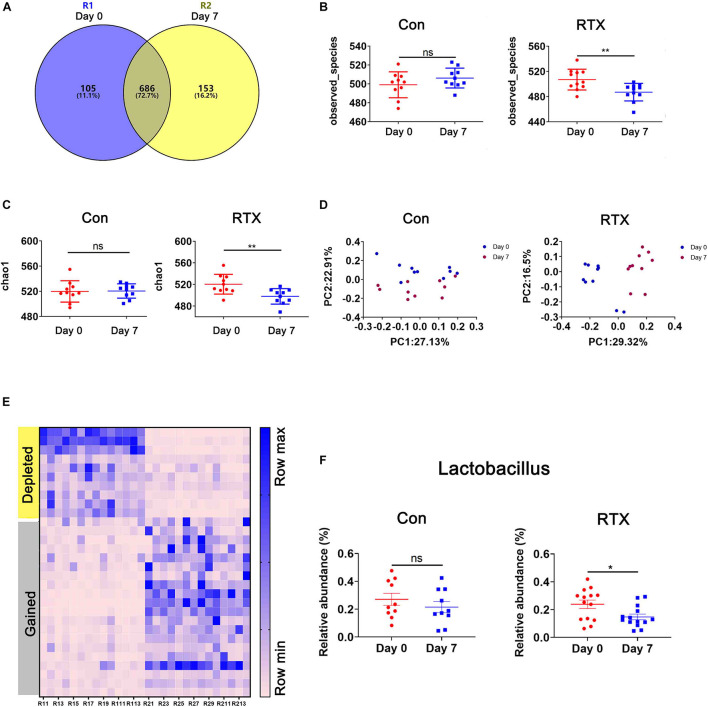
Rituximab treatment changes the composition of the intestinal microbiota. **(A)** The number of OTUs in the intestine of RTX-treated mice on day 0 (blue), day 7 (yellow), or shared on days 0 and 7 (gray). **(B,C)** Changes in the α diversity of the intestinal microbiota in the RTX-treated and control groups are shown on days 0 and 7. **(D)** The results of principal component analysis (PCA) of intestinal microbiota in the RTX-treated group and control group on days 0 and 7. **(E)** Heatmap of OTUs of intestinal microbiota in RTX-treated mice on days 0 and 7. **(F)** The relative abundance of 2 examples of OTUs in RTX-treated mice and control mice. OTUs, operational taxonomic units. *p < 0.05, **p < 0.01.

Recent studies have shown that some drugs cause changes in the intestinal microbial composition, such as cyclophosphamide and carboplatin. In addition, the intestinal microbiota is closely related to drug resistance, efficacy, and toxicity (Alexander et al., 2017; Bullman et al., 2017; Gopalakrishnan et al., 2018; Matson et al., 2018; Routy et al., 2018; Zhou et al., 2018). To determine whether RTX has an effect on the intestinal flora of mice, 16S ribosomal DNA (rDNA) sequencing was used, collecting stool samples before and after RTX treatment for bacterial microbiota profiling. RTX treatment changed the microbial population in mice (Figure 3A). Although most OTUs were shared on day 0 and day 7, 153 OTUs were lost, 105 unique OTUs appeared after RTX treatment, and the total number of OTUs decreased. Next, we analysed the bacterial alpha diversity in the RTX treatment and control groups. We found that the total microbial diversity decreased significantly in the RTX treatment group, while no significant change was observed in the control group over time (Figures 3B,C). Moreover, principal component analysis (PCA) revealed that the composition of the intestinal microflora changed significantly after RTX treatment (day 7) compared with before RTX treatment (day 0) (Figure 3D). In the control group, there was little difference in intestinal microbiota composition between days 7 and 0 (Figure 3D). In addition, significant changes in the intestinal microbiota were estimated by comparing the composition of OTUs with that of normal mice. Figure 3E shows the increase or decrease in microbial components in response to different treatments. For example, the relative abundance of Lactobacillus decreased significantly (Figure 3F), while there was no significant change in these OTUs between day 0 and day 7 in the control group. In conclusion, these data suggest that RTX treatment alters the diversity and composition of the intestinal microbiota in mice (the comparison of RTX and control groups about the results of α and β diversity on day 0 and day 7 was shown in Supplementary Figure 4).

### Identification of Key Microbial Species

**FIGURE 4:**
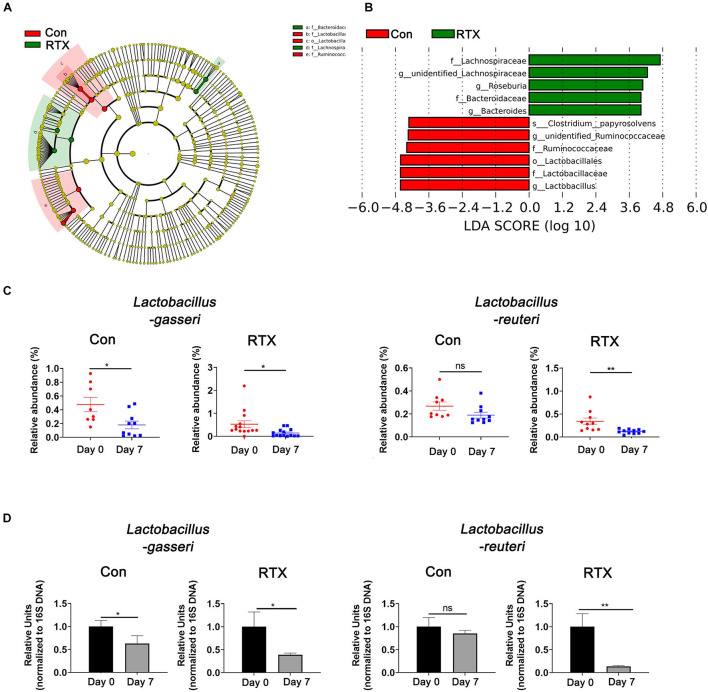
*Lactobacillus reuteri* is associated with RTX-induced intestinal mucositis. **(A)** LEfSe analysis of differentially abundant species in RTX-treated mice on day 0 (R1) and day 7 (R2). **(B)** LDA scores of differentially abundant species in the intestine before and after RTX treatment showing only the groups with an LDA threshold > 3.6. **(C)** The relative abundance of significantly different bacteria in control and RTX treatment groups. **(D)** qPCR results of different bacteria in feces samples. Data are shown as the mean ± SEM. *p < 0.05, **p < 0.01.

To identify the most differentially abundant bacteria after RTX treatment, LDA effect size analysis (LEfSe) was performed, and we selected biomarkers with LDA scores > 4. We found that Lactobacillus was more abundant before RTX administration and decreased after 7 days (Figures 4A,B). We further analysed the relative abundance of different Lactobacillus species in the control and RTX treatment groups. The results showed that the most differentially abundant bacteria were *Lactobacillus gasseri* (*L. gasseri*) in the RTX treatment group, consistent with the control group (Figure 4C). Only *L. reuteri* decreased significantly in the RTX-treated group, while no significant change was observed in the control group. These results demonstrate that the number of *L. reuteri* decreased significantly after RTX treatment compared with the control group. *L. reuteri* exhibits strong adhesion to the intestinal mucosa. It improves the distribution of intestinal microbiota, inhibits the colonization of harmful bacteria and resists intestinal diseases. We conducted qPCR to quantify the number of specific Lactobacillus in feces samples. After RTX treatment, the amount of *L. gasseri* decreased, but the number in the control group also decreased, while the number of *L. reuteri* did not decrease after PBS treatment (Figure 4D), consistent with previous reports. It should be noted that the changes in *L. reuteri* after RTX treatment were consistent with the 16S rDNA sequencing results, suggesting that *L. reuteri* may play a role in RTX-induced intestinal mucositis.

### *Lactobacillus reuteri* Inhibits Inflammation *in vitro*

**FIGURE 5:**
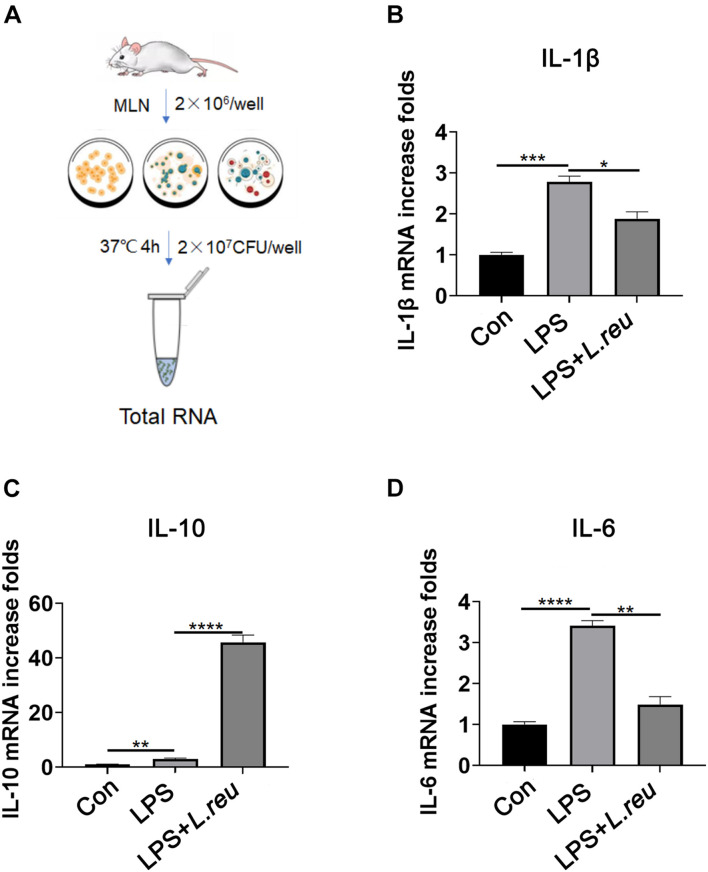
Effect of *Lactobacillus reuteri* on the expression of IL-1β, IL-10, and IL-6 in mesenteric lymph node cells stimulated by LPS *in vitro*. **(A)** Schematic diagram of experiment setup. In the presence or absence of *L. reuteri*, MLN cells were stimulated with LPS alone for 4 h. Cells were collected for qPCR analysis. **(B–D)** mRNA expression of IL-1β, IL-10, and IL-6 in MLN cells. Data are expressed as the mean ± SEM. *p* < 0.05, **p < 0.01, ***p < 0.001, ****P < 0.0001. Con, control; *L. reu*, *Lactobacillus reuteri*.

To invaluated the role of *L. reuteri* in RTX-induced intestinal mucositis. Next, we evaluated whether *L. reuteri* inhibits inflammation *in vitro* (Figure 5A). Mesenteric lymph node cells of mice were cultured *in vitro* and treated with *L. reuteri* or *L. reuteri* + LPS to assess the expression of inflammatory cytokines. Inflammatory cytokine expression was increased after LPS stimulation, but the expression of inflammatory cytokines in the MLN was not upregulated in the group coincubated with *L. reuteri* (Figures 5B–D). These results suggest that *L. reuteri* inhibits inflammatory reactions *in vitro* to some extent.

### *Lactobacillus reuteri* Attenuates Rituximab-Related Inflammatory Damage

**FIGURE 6:**
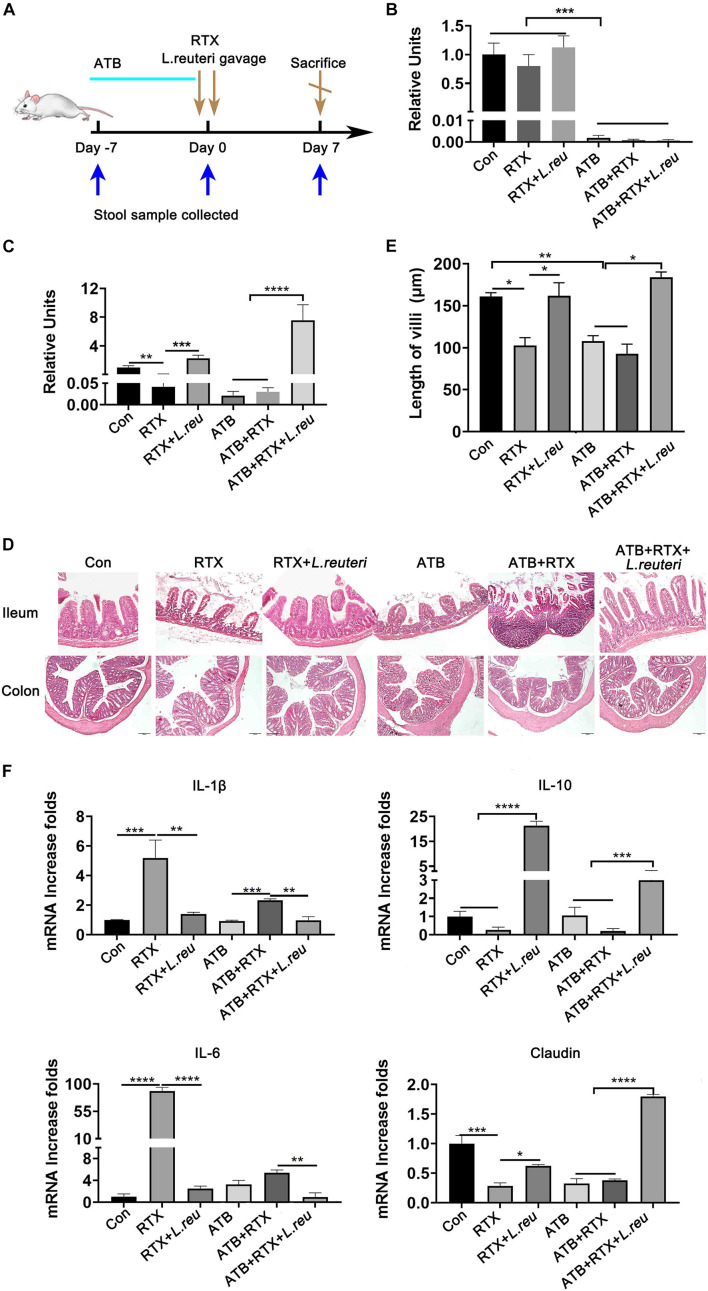
*Lactobacillus reuteri* attenuates RTX-induced inflammatory injury. **(A)** Experimental schedule. **(B)** Relative abundance of *L. reuteri* in the intestine of mice detected by qPCR after 5 days of antibiotic administration. **(C)** Relative abundance of *L. reuteri* in the intestine detected by qPCR after 5 days of *L. reuteri* gavage. **(D)** H&E staining of the ileum and colon (magnification, 40×). **(E)** Villus length of the ileum. **(F)** mRNA expression levels of IL-1β, IL-6, IL-10, and claudin in the ileum of mice. Data are expressed as the mean ± SEM. *p* < 0.05, **p < 0.01, ***p < 0.001, ****P < 0.0001. Con, control; ATB, antibiotic treatment; *L. reu*, *L. reuteri*.

**FIGURE 7:**
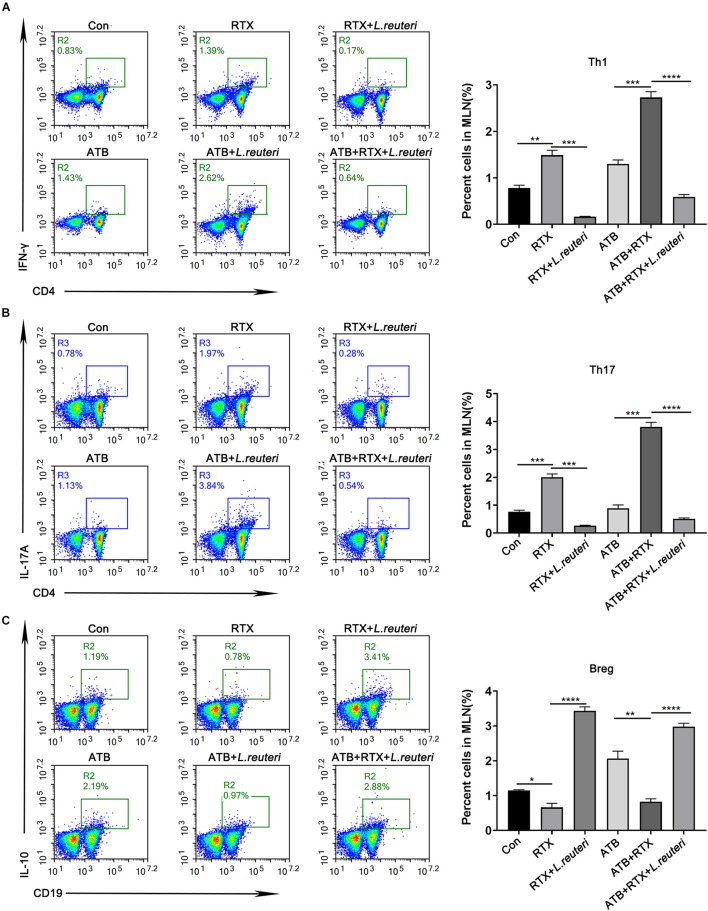
*Lactobacillus reuteri* supplementation alleviates Th cell imbalance induced by RTX and inhibits Th1 and Th17 immune responses. **(A)** Th1 cells, **(B)** Th17 cells, and **(C)** Breg cells in MLN cells. Data are expressed as the mean ± SEM. *p* < 0.05, **p < 0.01, ***p < 0.001, ****P < 0.0001.

It has been reported that *L. reuteri* reduces inflammatory reactions and relieves the development of colitis (Madsen et al., 1999; Liu et al., 2012; Mu et al., 2017, 2018). To verify whether the loss of intestinal *L. reuteri* leads to more severe inflammatory damage, colonic colonization was performed in antibiotic-treated mice with RTX by oral administration of *L. reuteri* (Figure 6A). After antibiotic treatment (day 0), levels of *L. reuteri* in the ATB, ATB + RTX, and ATB + RTX + *L. reuteri* groups were significantly decreased compared to those in the other three groups (Figure 6B). On day 5 after intragastric administration of *L. reuteri*, the *L. reuteri* content between the RTX + *L. reuteri* and ATB + RTX + *L. reuteri* groups was significantly higher than in the ATB, RTX, and ATB + RTX groups (Figure 6C). These results indicate that *L. reuteri* has a strong intestinal colonization effect in mice. Consistent with previous reports that *L. reuteri* loss exacerbates colitis, mice treated with RTX for 7 days developed more severe intestinal mucositis, in contrast to the control group, villous atrophy and intestinal epithelial injury of mice in the RTX treatment group were obvious, as shown in Figure 6D. Moreover, obvious inflammatory cell infiltration was apparent in the ileum tissue of ATB and RTX-treated mice (Figure 6D) as assessed by histological analysis of increased epithelial damage (Figure 6D) and shortened intestinal villi of ileum (Figure 6E), which resulted in a reduced mucositis score (Supplementary Figure 2) compared with the RTX-treated control group (Figure 6E). In addition, compared with RTX-treated mice, *L. reuteri*-colonized mice exhibited decreased levels of inflammatory cytokines (Figure 6F) and increased levels of intestinal tight junction proteins (Figure 6F). These findings suggest that *L. reuteri* alleviates RTX-induced intestinal mucositis and reduces damage to the intestinal mucosal barrier system. Next, we analysed changes in the immune cell population 7 days after *L. reuteri* was implanted. Compared with the control group, the number of Th1 and Th17 cells in the MLN of RTX-treated mice was significantly increased (Figure 7A). Consistent with our previous results, RTX treatment resulted in a significant increase in Th1 and Th17 cells in the MLN (Figures 7A,B). However, the number of Th1 and Th17 cells in the MLN of mice fed *L. reuteri* was significantly reduced, and Th2 cells was increased (Supplementary Figure 5). In addition, RTX treatment resulted in a decrease in Breg cells, and intragastric administration of *L. reuteri* resulted in an increase in Treg cells (Supplementary Figure 5) Breg cells in the MLN (Figure 7C). These results suggest that *L. reuteri* could alleviate the intestinal inflammation induced by RTX through regulating the amount of Th1 and Th17 cells.

## Discussion

The toxicity of many anticancer drugs seriously affects their therapeutic effects and the quality of life in cancer patients (El Fassi et al., 2008; Agathocleous et al., 2010; Ardelean et al., 2010; Blombery et al., 2011; Sekkach et al., 2011; Lipka et al., 2016; Ziȩtarska et al., 2017; Maestá et al., 2018; Shahmohammadi et al., 2018; Kaegi et al., 2019). Some drug treatments lead to intestinal mucosal dysfunction, including endothelial and epithelial cell death and even mucosal immune system activation (Chaveli-López and Bagán-Sebastián, 2016; de Melo Manzi et al., 2016; Shimamura et al., 2018). At present, there is no successful method to treat mucositis. In recent years, studies have shown that the intestinal microbiota regulates the therapeutic effect of some anticancer drugs (Authors et al., 2015; Sivan et al., 2015; Routy et al., 2018), but little is known about the regulatory function of the intestinal flora in the gastrointestinal toxicity of these drugs (Zhou et al., 2018; Yu et al., 2019). The intestinal mucosa acts as a selective barrier between the body and the external environment that blocks harmful substances from entering the systemic circulation but also ensures the absorption of nutrients (Isaacs-Ten et al., 2020). The intestinal microbiota lives on the layer of mucus secreted by intestinal goblet cells. The intestinal microbiota is involved in the regulation of mucosal barrier function, immune balance, prevention of pathogen infection, vitamin synthesis and metabolism (Gibson, 2009).

Our results suggest that the decrease in *L. reuteri* may be related to RTX-induced mucositis. Previous studies have shown that the absence of *L. reuteri* leads to colic in infants, *L. reuteri* supplementation alleviates the damage of DSS-induced colitis in mice, and *L. reuteri* can be used to treat enteritis in IL-10-deficient mice (Madsen et al., 1999; Liu et al., 2012; Bene et al., 2017; Mu et al., 2017, 2018; Savino et al., 2018). Other studies have shown that *L. reuteri* may help to alleviate chronic inflammation (Madsen et al., 1999; Liu et al., 2012; Mu et al., 2017, 2018). All these studies have illustrated a close relationship between *L. reuteri* and inflammation.

We used antibiotics to reduce the abundance of *L. reuteri* in the intestine of mice. We found that RTX induced intestinal mucosal injury and aggravated inflammatory reactions, with marked inflammatory cell infiltration. However, our combined antibiotics were broad-spectrum antibiotics that are mainly used for anaerobic bacteria that not only remove *L. reuteri* but also reduce the content of many intestinal microbiota (Routy et al., 2018; Zhou et al., 2018; Yu et al., 2019), which does not exclude the role of these microbiota in the process of RTX-induced mucositis. Therefore, we designed an additional experimental control group to study the effect of *L. reuteri* on mucositis. The results revealed that *L. reuteri* supplementation alleviates intestinal tissue injury and the inflammatory response induced by RTX. Detection of Claudin-1 in intestinal tissue showed that the damaged intestinal mucosal barrier exhibited signs of repair. These results suggest that *L. reuteri* alleviates RTX-induced intestinal mucositis. However, the intestinal microbial community is a complex system, and its stability is closely related to the normal digestion, metabolism and immune regulation of the host. The occurrence of intestinal mucositis is not limited to the influence of a single strain but also involves the network regulation mechanism of the formation of other strains or metabolites. The relationship between other strains and RTX-induced intestinal mucositis needs further study. Previous studies have described the mechanism by which the intestinal microbiota regulates the efficacy of anticancer drugs, which can be summarized as the framework of transport, immune regulation, metabolism, enzyme degradation, and diversity reduction (Alexander et al., 2017; Roy and Trinchieri, 2017). A previous study reported that chemotherapy drugs lead to the destruction of intestinal structure, accompanied by the transfer of some symbiotic bacteria to the secondary lymphoid organs of mice, stimulating the response of Th1 and Th17 cells, and the antitumor effect of the drug was weakened after antibiotic treatment (Viaud et al., 2013; Yu et al., 2019). These data suggest that intestinal microbial translocation caused by intestinal mucosal damage induces an immune response and affects the efficacy of drugs. In this study, RTX caused intestinal atrophy, infiltration of inflammatory cells and destruction of the intestinal mucosal barrier. Therefore, we speculate that RTX-induced intestinal damage may lead to an imbalance in the intestinal microbiota and trigger an immune response. Results demonstrated that Th1 and Th17 cells in MLNs were significantly increased in response to RTX treatment. However, after *L. reuteri* supplementation, Th cells decreased while Breg cells increased in MLNs. Therefore, *L. reuteri* may alleviate the gastrointestinal toxicity induced by RTX through Th cells.

Regulatory B cells (Bregs) is a kind of immunosuppressive cells, which can inhibit a variety of immunopathology by producing cytokines such as interleukin-10 (IL-10). Luu et al.’s (2019) research shows that the anti-inflammatory effect of SCFA (especially pentanoate) was not only reflected in strong upregulation of IL-10 production in Bregs but also by a potent suppression of pathogenic Th17 cell phenotype. Yan et al. showed that *L. reuteri* produce short chain fatty acids (SCFAs) as a major fertilization product (Stewart et al., 2009; Den Besten et al., 2013; Furusawa et al., 2013). In addition, Nagpal et al. (2018) and others believe that the SC FA content in the intestine will increase after *L. reuteri* supplementation. It is speculated that *L. reuteri* may affect the content of Bregs by regulating the metabolism of SCFA. However, the specific mechanism still needs to be further explored.

We demonstrated that *L. reuteri* alleviates the upregulation of inflammatory cytokines in MLN cells stimulated by LPS *in vitro*. Therefore, *L. reuteri* alleviates the inflammatory stimulation caused by RTX and inhibits the local and systemic immune response caused by intestinal mucosal damage in response to RTX. Although this study shows some promising results, it also has limitations. RTX is an anticancer drug widely used in non-Hodgkin’s lymphoma. It is also related to changes in the intestinal flora. We should conduct more experiments in cancer models not limited to normal mice to verify the relationship between the gut microbiota and RTX treatment. In conclusion, our data suggest that the regulation of intestinal microbiota may be a new way to reduce the toxicity of anticancer drugs.

## Data Availability Statement

The datasets presented in this study can be found in online repositories. The names of the repository/repositories and accession number(s) can be found in the article/Supplementary Material.

## Ethics Statement

The animal study was reviewed and approved by the Animal Protection Committee of Sichuan University (Sichuan, China).

## Author Contributions

LY, BaZ, and BiZ contributed to conception and design of the study. BaZ organized the database. CD performed the statistical analysis. BiZ wrote the first draft of the manuscript. RZ, DX, and YT wrote sections of the manuscript. All authors contributed to manuscript revision, read, and approved the submitted version.

## Conflict of Interest

The authors declare that the research was conducted in the absence of any commercial or financial relationships that could be construed as a potential conflict of interest.

## Publisher’s Note

All claims expressed in this article are solely those of the authors and do not necessarily represent those of their affiliated organizations, or those of the publisher, the editors and the reviewers. Any product that may be evaluated in this article, or claim that may be made by its manufacturer, is not guaranteed or endorsed by the publisher.

## Footnotes

1
http://ccb.jhu.edu/software/FLASH/

2
http://qiime.org/scripts/split_libraries_fastq.html

3
https://www.arb-silva.de/

4
http://www.drive5.com/usearch/manual/uchime_algo.html

5
http://drive5.com/uparse/

6
http://www.drive5.com/muscle/
