# Supplementary material for: Lactobacillus reuteri Alleviates Gastrointestinal Toxicity of Rituximab by Regulating the Proinflammatory T Cells in vivo
Source: Front Microbiol. 2021 Oct 12;12:645500. doi: 10.3389/fmicb.2021.645500 (PMC8546249; doi:10.3389/fmicb.2021.645500)
Supplement: Supplementary file 1 [file Data_Sheet_1.docx]

Supplementary Material

## Supplementary Figures


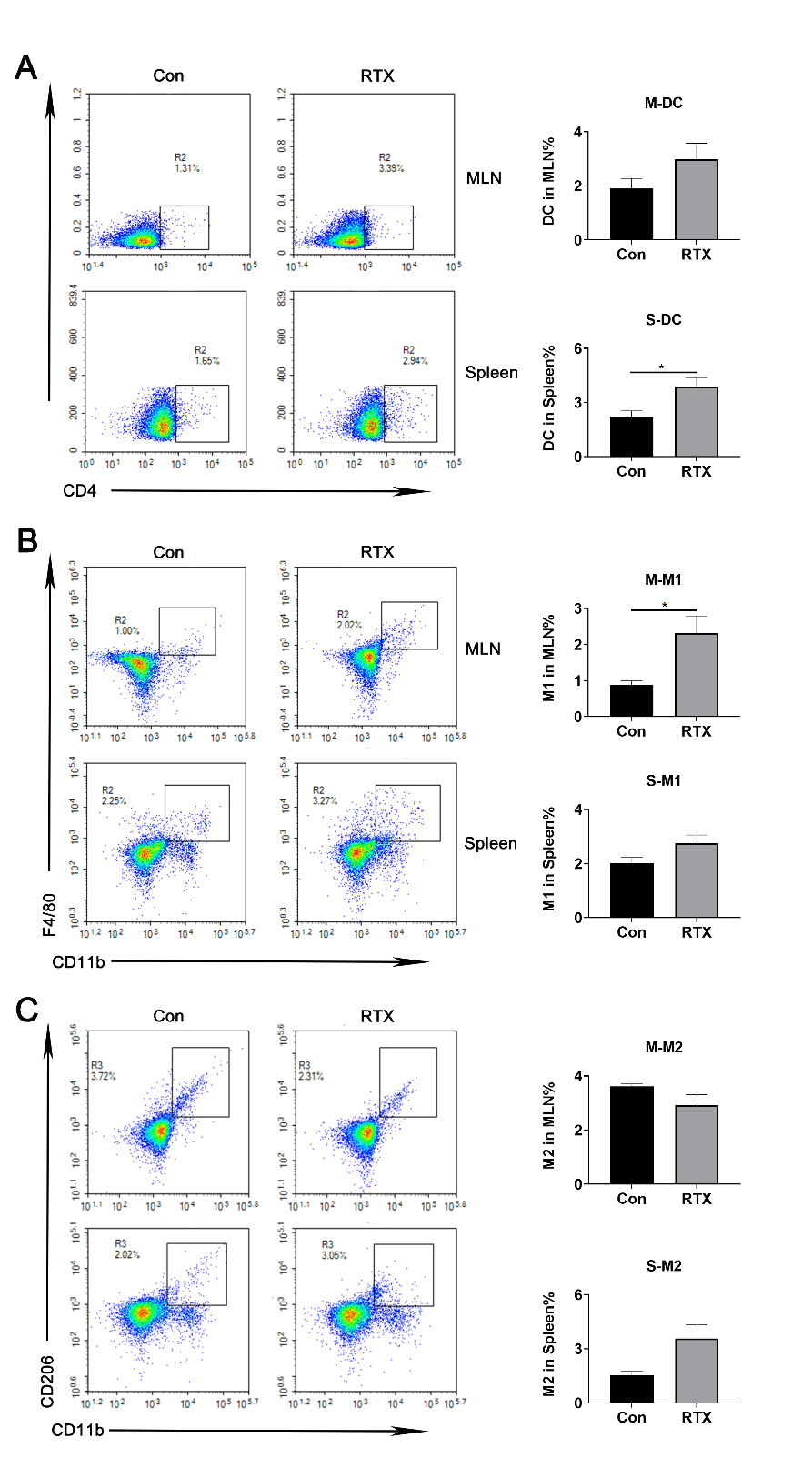


**Fig. S1: Changes in macrophage cells induced by RTX.** The percentages of A. CD4^+^ cells, B. CD11b^+^ F4/80^+^ and C. CD11b^+^ CD206^+^ macrophages in the spleen and MLN were detected by flow cytometry. Data are expressed as the mean ± SEM. **p* < 0.05.


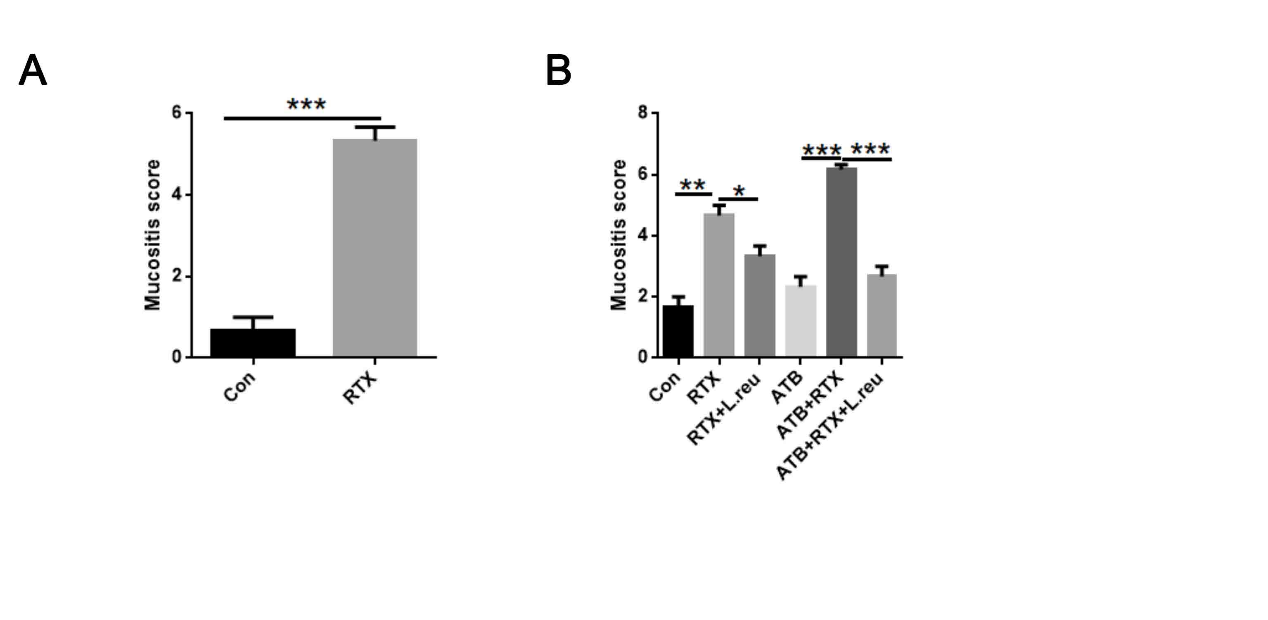


**Fig. S2:** Under the microscope, the small intestine slices stained with H&E were photographed with a magnification of 40. Image J software was used to measure the villus length of ileum, and statistical analysis was carried out. The severity of intestinal mucositis was evaluated according to the indexes of intestinal mucosal necrosis, inflammatory infiltration and villous atrophy: normal (0), mild (1), moderate (2) and severe (3). The sum of different indexes of a single sample was the score of mucositis. A-B. Histological mucositis scores for the small intestine. p < 0.05, ** p < 0.01, *** p < 0.001.


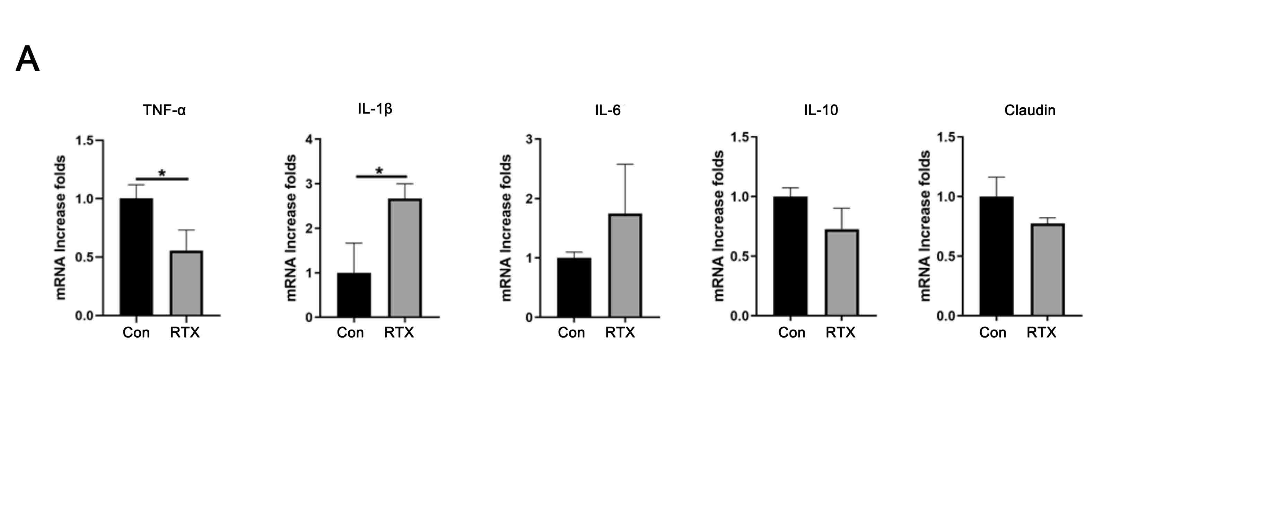


**Fig. S3: The expression of some cytokines in colon before and after RTX treatment.** A. Expression levels of TNF-α, IL-1β, IL-6, IL-10 and claudin in the colon of mice treated with RTX or normal saline. Data are expressed as the mean ± SEM. *p<0.05.


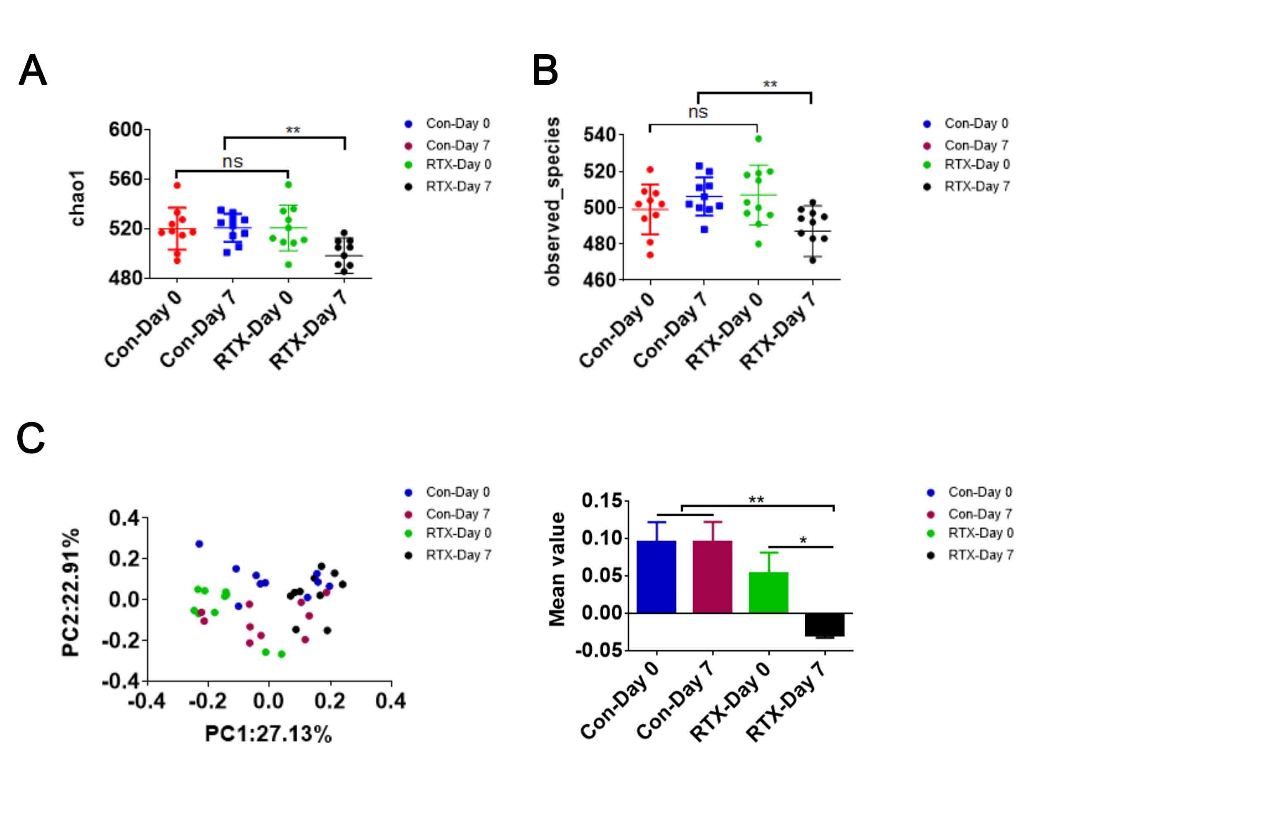


**Fig. S4: RTX treatment changes the composition of the intestinal microbiota.** A-B. Changes in the α diversity of the intestinal microbiota in the RTX-treated and control groups are shown on days 0 and 7. C. The results of principal component analysis (PCA) of intestinal microbiota in the RTX-treated group and control group on days 0 and 7 in the left panel. And the P value was calculated by using column statistics in GraphPad software. p < 0.05, ** p < 0.01.


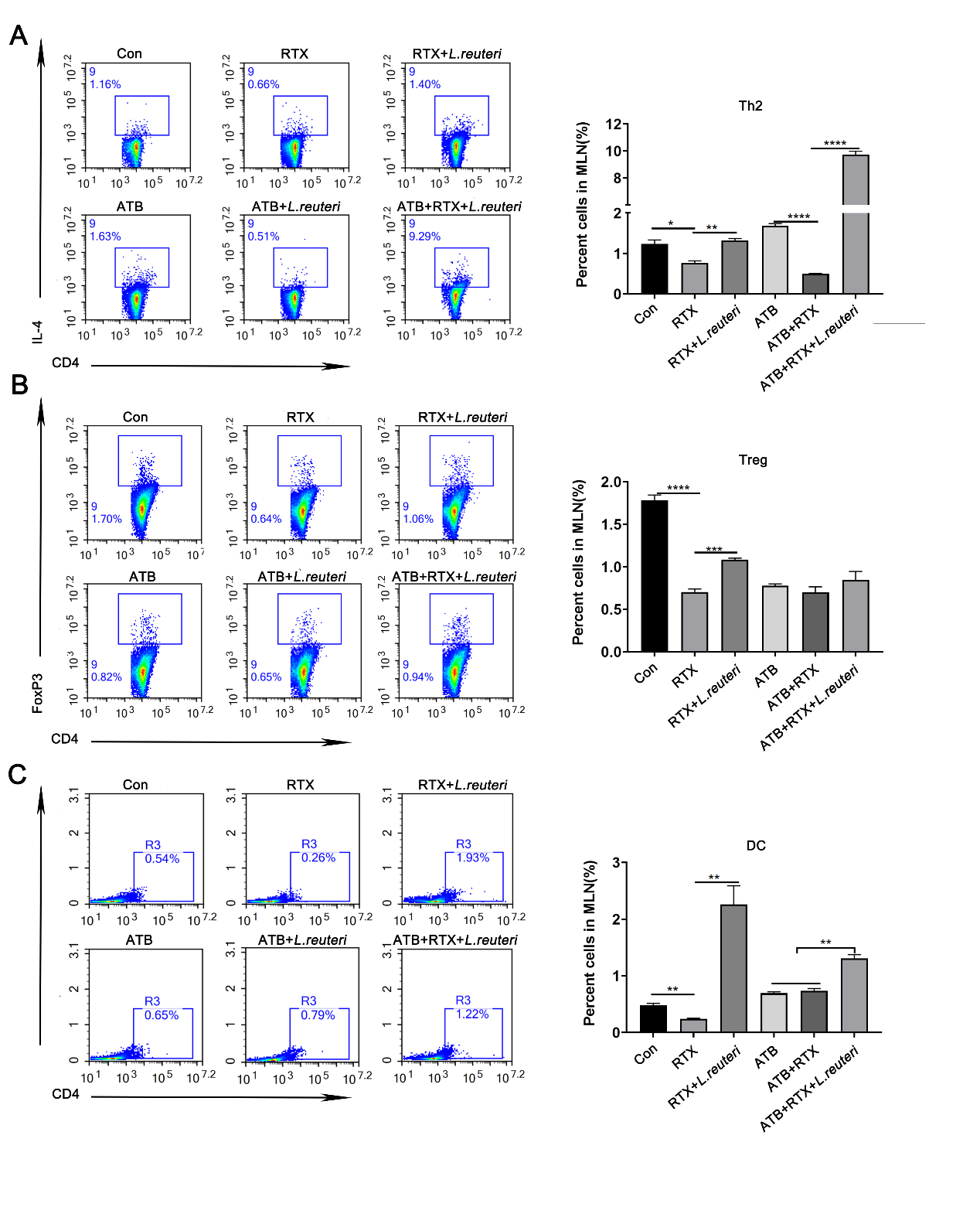


**Fig. S5:** *L. reuteri* **gavage alleviated the imbalance of Th cells induced by RTX and affected the immune responses of Th1 and Th17 cells.** Percentages of Th2 cells, Treg cells and DC cells detected in MLN cells. Data are expressed as the mean ± SEM. p < 0.05, ** p < 0.01, *** p < 0.001, **** P < 0.0001.
